# Sensitive Detection of Sulfide Ion Based on Fluorescent Ionic Liquid–Graphene Quantum Dots Nanocomposite

**DOI:** 10.3389/fchem.2021.658045

**Published:** 2021-04-30

**Authors:** Guanhua Qiu, Yaoqi Han, Xiaoqi Zhu, Jiawei Gong, Tao Luo, Chang Zhao, Junjie Liu, Jiyang Liu, Xiang Li

**Affiliations:** ^1^ Guangxi Medical University Cancer Hospital, Guangxi Medical University, Nanning, China; ^2^ Central Hospital Affiliated to Shandong First Medical University, Jinan, China; ^3^ School of Basic Medicine, Guangxi Medical University, Nanning, China; ^4^ Department of Chemistry, Zhejiang Sci-Tech University, Xiasha Higher Education Zone, Hangzhou, China

**Keywords:** fluorescent sensor, sulfide ion, ionic liquid, graphene quantum dots, nanocomposite

## Abstract

Sulfide ions (S^2−^) that are widely distributed in biological and industrial fields are extremely toxic and pose great harms to both ecological environment and human health. However, fluorescent sensors toward S^2−^ ions commonly use S^2−^-recovered fluorescence of fluorophore that is first quenched mainly by metal ions. Fluorescent probe which enables direct, selective, and sensitive detection of S^2−^ ion is highly desirable. Herein, we demonstrate one-step preparation of fluorescent ionic liquid–graphene quantum dots (IL-GQDs) nanocomposite, which can act as a fluorescent probe for direct and sensitive detection of S^2−^ ion. The IL-GQDs nanocomposite is easily synthesized *via* facile molecular fusion of carbon precursor and *in situ* surface modification of GQDs by IL under hydrothermal condition. The as-prepared IL-GQDs nanocomposite has uniform and ultrasmall size, high crystallinity, and bright green fluorescence (absolute photoluminescence quantum yield of 18.2%). S^2−^ ions can strongly and selectively quench the fluorescence of IL-GQDs because of the anion exchange ability of IL. With IL-GQDs nanocomposite being fluorescent probe, direct and sensitive detection of S^2−^ is realized with a linear detection range of 100nM–10μM and 10μM–0.2mM (limit of detection or LOD of 23nM). Detection of S^2−^ ions in environmental river water is also achieved.

## Introduction

Sulfide ions (S^2−^) are crucial and widely distributed in many biological systems and industrial fields (Gao et al., 2017; Gong et al., 2017; Wang et al., 2017). For instance, S^2−^ ions participate in mitochondrial electron transport chain (ETC) in cell, and some microbes in water can produce S^2−^ through reduction of sulfate ions. At the same time, S^2−^ ions are widely used and released in industrial processes such as oil refineries, paper mills, and sewage treatment. However, the high toxicity of S^2−^ ion poses great harm to both ecological environment and human health (Barati et al., 2016). For humans, long-term exposure to low concentrations of S^2−^ ions can cause chronic diseases in blood, eyes, skin, and digestive and respiratory systems. In addition, high concentrations of S^2−^ ion results in serious human neurological diseases (Barati et al., 2016; Gao et al., 2017; Gong et al., 2017; Wang et al., 2017). Therefore, the concentration of S^2−^ ions has been an important index in the field of biological, environmental, and health systems. Simple, fast, and sensitive detection of S^2−^ ion is highly desirable.

So far, S^2−^ ion detection technologies based on electrochemical sensors, ion chromatography, or colorimetry have been reported (Ni et al., 2015; Barati et al., 2016; Chen et al., 2016). Among them, fluorescent sensor has attracted great attention because of its simple operation, fast detection speed, and high sensitivity (Yu et al., 2015; Luo et al., 2019; Cui et al., 2020). Unlike the commonly reported fluorescent sensors for the detection of cations (such as metal ions), the detection of S^2−^ ion mainly employs the recovery of fluorophore’s fluorescence that is first quenched by a metal ion (Bhopate et al., 2015; Liu et al., 2016). For example, the fluorescence of curcumin nanoparticles can be quenched by Cu^2+^ ion, and the quenched fluorescence can be recovered by the added S^2−^ ion (Bhopate et al., 2015). Thus, fluorescent detection of S^2−^ ions is mainly based on fluorescence off–on strategy. Facile synthesis of fluorescent probe with direct selectivity toward S^2−^ ion is of great significance but remains as a huge challenge.

Graphene quantum dots (GQDs) or zero-dimensional (0D) graphene are the latest high-value addition to the family of carbon nanomaterials (Li et al., 2016a; Li et al., 2016b; Yan et al., 2018; Yan et al., 2019). As planar and 0D nanocarbons with atomic layer thickness, nanometer wide, and sp (Wang et al., 2017) carbon domain, GQDs have unique characteristics (Ali and Hassan, 2021; Kadian et al., 2021; Pierrat and Gaumet, 2021). For example, their ultrasmall size leads to abundant active edge sites and excellent water dispersibility (Lu et al., 2018; Zhao et al., 2020). In addition, the opening of the bandgap leads to the fluorescence of GQDs. The unique merits including molecular size, highly adjustable chemical properties, easy functionalization, and good dispersibility endow GQDs with great potential in the field of fluorescence sensing. However, like other fluorescent probes, GQDs are often used for cation detection. It is necessary to adjust the structure and property of GQDs to promote their application in direct anion sensing.

In addition to GQDs doped with heteroatoms or modified with functional groups, GQD-based functional nanocomposite also has great potential to introduce new function. Ionic liquid (IL) that is composed of organic cations and inorganic anions, and exists as a liquid at room temperature has great potential for the preparation of functional fluorescent nanocarbons. For example, IL could be used as the precursor or modifier to prepare functional carbon dots (CDs), carbon nanoparticles (CNPs), or carbon nanoribbons (Xiao et al., 2013; Wang et al., 2015; Liu et al., 2017; Shu et al., 2017; Sun et al., 2017; Zhuo et al., 2017; Pham-Truong et al., 2018; Tang et al., 2018). It is worth noting that IL-modified materials possess capability of anion exchange (Wang et al., 2015; Sun et al., 2017; Luo et al., 2019). Taking advantages of the variability of structure and properties of IL and nanocarbon (Das G. S. et al., 2019; Das G. S. et al., 2019; Tripathi et al., 2020), the possibilities of IL–nanocarbon composites for direct sensing of anions can be further expanded. However, the reported methods to introduce IL in nanocarbon usually suffer from harsh conditions (e.g., sulfuric acid carbonization and high temperature beyond normal operation), accurate control (e.g., potential of electrolyte in electrochemical exfoliating), long time (e.g., two runs of dialysis in post-modification), and low production yield.

**FIGURE 1:**
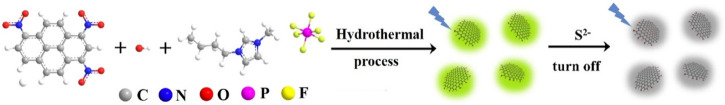
Schematic illustration for one-step preparation of fluorescent IL-GQDs nanocomposites and their application for detection of S^2−^ based on fluorescence turn off.

In this work, fluorescent nanocomposite based on IL-modified GQDs (IL-GQDs) is simply synthesized, which enables sensitive detection of S^2−^ ions. As illustrated in Figure 1, IL-GQDs nanocomposite is synthesized through one-step bottom-up molecular fusion using 1,3,6-trinitropyrene as carbon a precursor and 1-butyl-3-methylimidazolium hexafluorophosphate ([BMIM][PF_6_]) as a modifier under hydrothermal condition. The synthesis of IL-GQDs nanocomposite is simple, fast, green, and scalable. The as-prepared IL-GQDs nanocomposite has bright fluorescence, high crystallinity, and uniform size. Owing to the anion exchange of [BMIM][PF_6_], S^2−^ ions can strongly and selectively quench the fluorescence of IL-GQDs nanocomposite. Sensitive detection of S^2−^ ions is demonstrated when IL-GQDs nanocomposite is employed as a fluorescent probe.

## Materials and Methods

### One-step Synthesis of IL-GQDs Nanocomposite

1,3,6-trinitropyrene was prepared as previously reported (Wang et al., 2014). IL-GQDs nanocomposite was synthesized using hydrothermal reaction in a ternary mixture of 1,3,6-trinitropyrene (2mg/ml), NaOH (0.1M), and [BMIM][PF_6_] (1% v/v) at 180C for 10h. The obtained solution was then dialyzed (cutoff molecular weight of 1,000Da) for 48h with stirring to completely remove unreacted small molecules. After filtering with a microporous membrane (0.22µm), IL-GQDs nanocomposite was obtained after lyophilization. For comparison, GQDs without IL modification (only with hydroxyl groups, OH-GQDs) were also synthesized using the same method but without the addition of IL. In addition, IL (1% v/v) was also treated at the same condition as the control.

### Characterization

Transmission electron microscopy (TEM) images of IL-GQDs were obtained on a JEM-2100 transmission electron microscope (JEOL Ltd., Japan; operating voltage: 200kV; supporting film: ultrathin carbon film). Atomic force microscopy (AFM) images of IL-GQDs were obtained using tapping mode on Bruker Multimode 8 (Bruker. Inc., United States; substrate: freshly exfoliated mica). X-ray photoelectron spectroscopy (XPS) was obtained from a PHI5300 electron spectrometer (PE, United States) with MgKá radiation (250W, 14kV). Fluorescence spectra, absolute photoluminescence quantum yields, and fluorescence lifetime were recorded on an FL 3C-11 spectrofluorometer (Hariba Scientific, United States). The X-ray powder diffraction (XRD) pattern of IL-GQDs was measured on a DX-2700 diffractometer (Dandong Haoyuan Instrument Co. Ltd., China).

### Fluorescent Detection of S^2−^ Ion

Effects of different anions and cations on the fluorescence of IL-GQDs were investigated. Different cations (Fe^3+^, Na^+^, Pb^2+^, K^+^, Cu^2+^, Cd^3+^, Al^3+^, Zn^2+^, Mg^2+^, Ca^2+^, Ag^+^, and Hg^2+^) or anions (S^2−^, HCO_3_^−^, HPO_4_^2−^, SO_4_^2−^, SCN^−^, I^−^,Cl^−^, NO_3_^−^, Ac^−^, NO_2_^−^, and Br^−^) were tested. Briefly, fluorescent intensity of IL-GQDs solution in the absence or presence of different single or mixed ions was measured. In case of mixed cations, ascorbic acid (AA, 20mM) was added to reduce the influence of iron ions. Two parameters including the relative fluorescence ratio (*F*/*F*_*0*_) or fluorescent quenching ratio (*F*_*0*_- *F*/*F*_*0*_) were used to evaluate the quenching of IL-GQDs caused by each ion, where *F*_*0*_ and *F* were the fluorescent intensity of IL-GQDs in the absence and presence of different ions, respectively.

Different concentrations of S^2−^ were obtained through stepwise dilution of the standard stock solutions (0.1M) in 4-(2-hydroxyethyl)-1-piperazineethanesulfonic acid (HEPES) buffer (0.1 M, pH 7.0, containing 20mM AA). After IL-GQDs reacted with different amounts of S^2−^ for 8min at room temperature, the fluorescent intensity of the solution was recorded (excitation wavelength: 470nm, emission wavelength: 520nm).

## Results and Discussion

### The Strategy for One-Step Synthesis of IL-GQDs Nanocomposite

So far, two different strategies have been developed to synthesize GQDs. One is the “top-down” method, that is, cutting large graphitized carbon materials including graphene, carbon black, or carbon nanotubes through chemical, electrochemical, or physical approaches. The other is the “bottom-up” method, which is mainly based on fusion of small organic molecules under hydrothermal/solvothermal condition or pyrolysis/carbonization of organic precursors at high temperatures. Compared with the top-down method, the bottom-up synthesis usually has higher production yield, narrower size distribution, and easy adjustment of structure and performance (e.g., by changing the carbon source, dopants, or modifiers). As shown in Figure 1, we use the one-step bottom-up method to synthesize IL-GQDs nanocomposite. The selected carbon precursor, 1,3,6-trinitropyrene, consists of four peri-fused benzene rings with a unique carbon skeleton like the primitive cell of graphene (Supplementary Scheme S1 in SI)(Wang et al., 2014). GQDs are formed through fusion of 1,3,6-trinitropyrene under hydrothermal conditions. Yan et al. (2020) used density functional theory (DFT) calculation to investigate the mechanisms underlying GQD growth through molecular fusion of polycyclic aromatic hydrocarbons. The precursor is dinitronaphthalene. It is revealed that under alkaline hydrothermal conditions, dinitronaphthalene undergoes dehydrogenation first, followed by denitration. Thus, the naphthalene alkyne with two dangling bonds sequentially conjugates with two other naphthalene alkyne molecules. The subsequent dehydrogenation and denitration of the resulting polyaromatic molecule allow similar growth. Continuous growth of GQDs leads to an increase of the total energy cost for dehydrogenation and denitration, causing the terminated fusion of precursor and self-limiting of the GQD size. Owing to the similar aromatic ring and nitro groups, we speculate that the formation of GQDs by molecular fusion of 1,3,6-trinitropyrene under alkaline hydrothermal conditions follows a similar mechanism. At the same time, the formed GQDs may exhibit strong non-covalent interaction with IL [BMIM][PF_6_], through π-π, cation-π, or electrostatic interactions. Thus, one-step synthesis of IL-modified GQDs is realized. The production yield of IL-GQDs nanocomposite is as high as 72% relative to the precursor used. According to the reports, one-step preparation of IL-modified nanocarbons (e.g., carbon dots, carbon nanoparticles, and carbon nanoribbons) usually involves harsh conditions such as sulfuric acid carbonization and pyrolysis at high temperature (such as 260–280 °C) beyond normal operation (usually 200°C for hydrothermal condition or 250°C for oven)(Liu et al., 2017; Sun et al., 2017; Zhuo et al., 2017). Thus, our method for the preparation of IL-GQDs is simple and green. Compared with IL-GQDs prepared through post-modification of hydroxyl-functionalized GQDs (OH-GQDs) with [BMIM][BF_4_] under ultrasonic treatment (Das G. S. et al., 2019) that contains two runs of dialysis, our one-step strategy is simple and time-saving.

### Characterization of IL-GQDs

**FIGURE 2:**
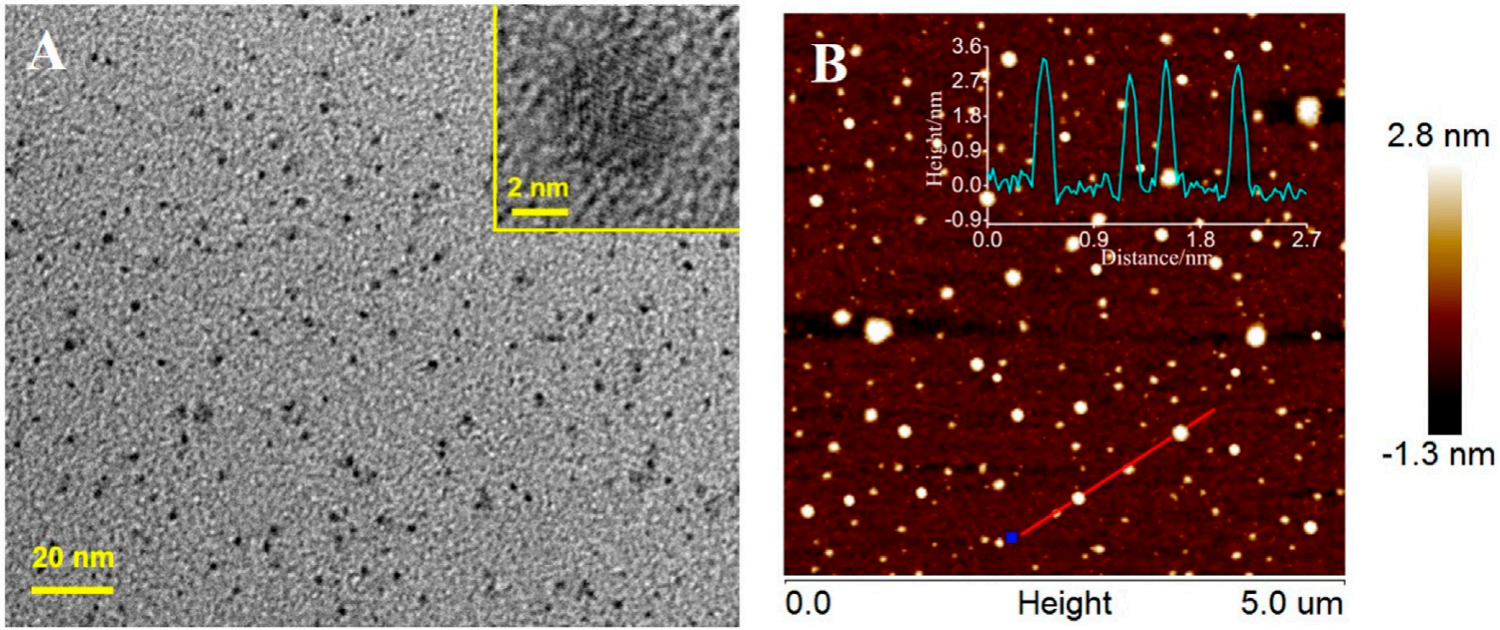
**(A)** TEM and HRTEM (insets) images of IL-GQDs. **(B)** AFM images of IL-GQDs. Inserted are the corresponding height profile along the indicated line.

Figure 2A shows a transmission electron microscopy (TEM) image of IL-GQDs. The IL-GQDs nanocomposite has a uniform and well-distributed structure with a diameter of about 2–3nm. The high-resolution TEM (HRTEM) image (Figure 2A inset) shows graphitic carbon with a lattice spacing of 0.21 nm, indicating (100) facet of graphene (Kwon et al., 2016). Atomic force microscopy (AFM) image indicates that the thickness of IL-GQDs nanocomposite is about 3.4nm (Figure 2B). Compared with the hydroxyl-modified GQDs (OH-GQDs) that are synthesized using the same procedure without adding IL (∼2.8nm) (Das G. S. et al., 2019), IL-GQDs nanocomposite exhibits higher thickness, indicating that GQDs are bound to IL. In the XRD pattern, IL-GQDs nanocomposite exhibits a broad interlayer (002) peak (Stobinski et al., 2014), which results from the few layer structure (Supplementary Figure S1 in SI).

**FIGURE 3:**
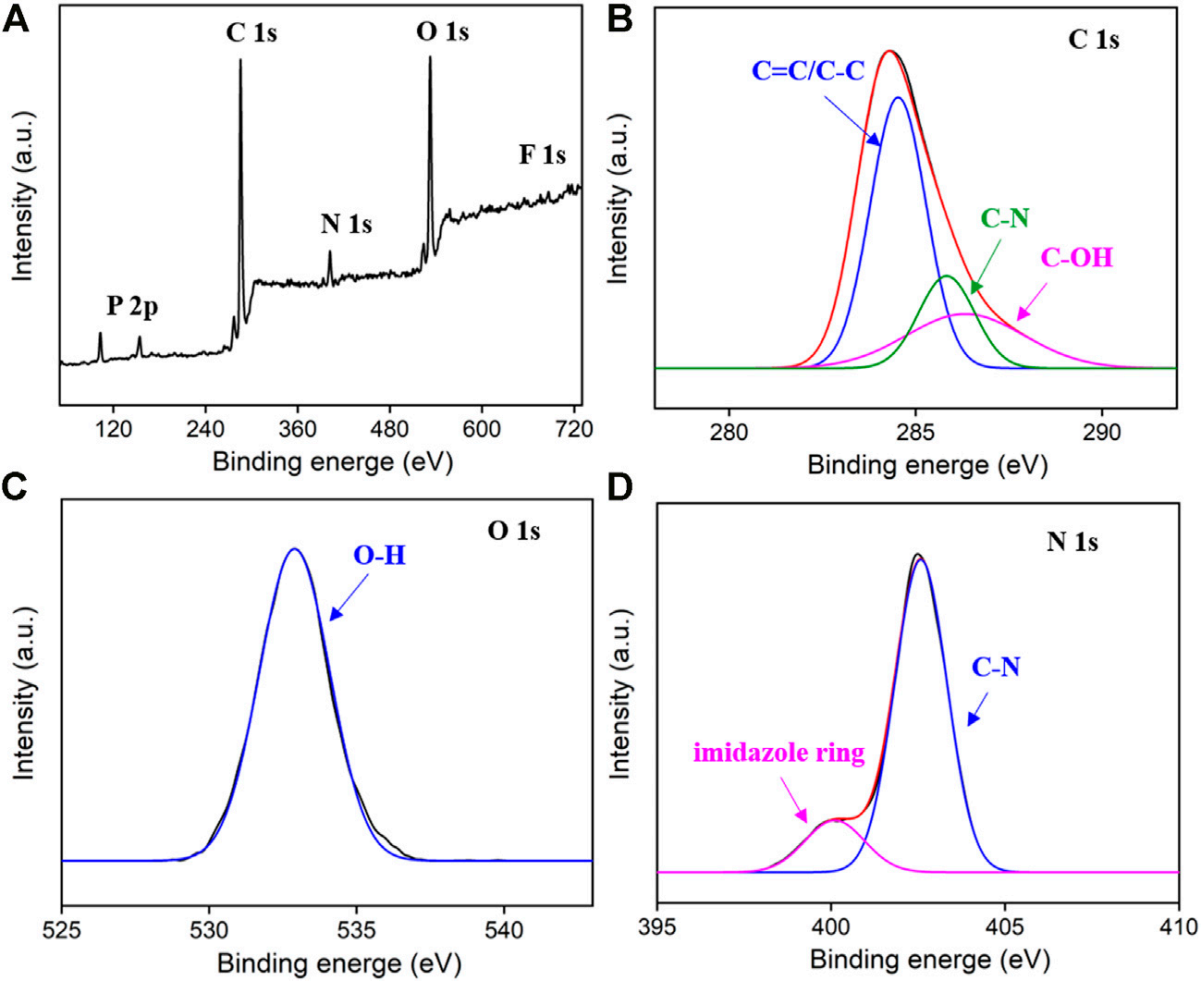
The XPS survey spectrum **(A)** and high-resolution spectra C1s, **(B)** O1s, **(C)** and N1s **(D)** of IL-GQDs.

X-ray photoelectron spectroscopy (XPS) is used to further study the chemical composition of the as-prepared IL-GQDs. As shown in Figure 3A, the XPS survey spectrum of IL-GQDs has remarkable signals of C, O, N, *p*, and F, indicating the composite between IL and GQDs. In the high-resolution C1s spectrum, the sp (Wang et al., 2017) structure of C=C of graphene (Figure 3B) verifies the crystallinity of IL-GQDs. The C-O structure in the C1s high-resolution spectrum (Figure 3B) and -OH peak in the O1s high-resolution spectrum (Figure 3B) indicate a large number of polar -OH groups (Figure 3C)(Wang et al., 2014). It is speculated that these -OH groups contribute to the good dispersibility of IL-GQDs. The appearance of imidazole ring and C-N peaks in high-resolution spectrum of N1s also confirms the existence of BMIM^+^ in IL-GQDs (Figure 3D). These evidences all prove that IL-GQDs are successfully formed in the one-step hydrothermal process. We speculate that the electrostatic interaction, cation-π interaction, and hydrophobic interaction are the reasons for the hybridization between IL and GQDs.

### Fluorescent Characteristics of IL-GQDs

**FIGURE 4:**
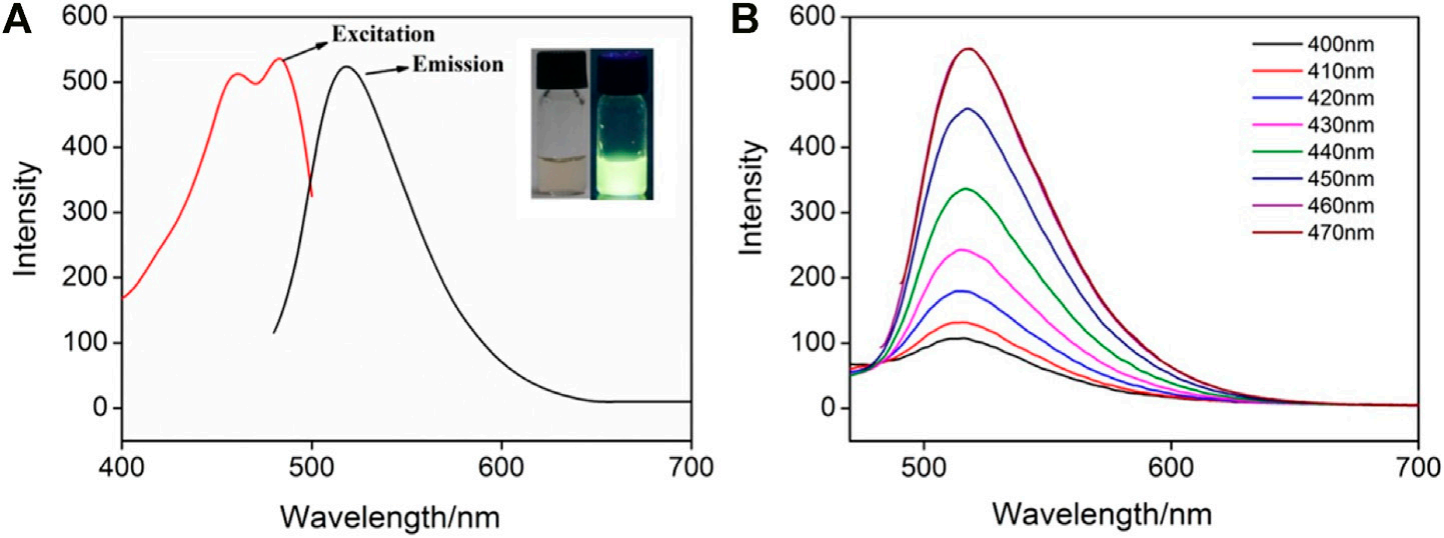
**(A)** Fluorescence (FL) excitation and emission spectra of IL-GQDs. Insets present the photographs of solutions under visible **(left)** or 365nm UV **(right)** light. **(B)** FL emission spectra of IL-GQDs under different excitation wavelength.

GQDs usually have fluorescence because of their ultrasmall size and the quantum confinement effect (Figure 4A). As shown, the IL-GQDs aqueous solution is light yellow under visible light and emits bright and green fluorescence under ultraviolet light (Figure 4A inset). On the contrary, no fluorescent materials could be obtained when only IL (1% v/v) is treated at the same condition (Supplementary Figure S2 in SI). Thus, GQDs are not formed by the applied IL. The maximum excitation wavelength and emission wavelength of IL-GQDs nanocomposite are 470 and 520nm, respectively. When the excitation wavelength increases from 400 to 470nm, the FL emission intensity of IL-GQDs increases with unchanged excitation wavelength, indicating that the fluorescence emission is independent of emission (Figure 4B). The absolute photoluminescence quantum yield (QY) of IL-GQDs is 18.2%. The fluorescence intensity of IL-GQDs at different pH was studied (Supplementary Figure S3A in SI). The pH of the solution is adjusted by adding sodium hydroxide or hydrochloric acid. In a strong acid solution, the fluorescence intensity increases sharply as the pH value increases. When the pH value is higher than 5, the fluorescence intensity is stable. This phenomenon may be attributed to the changes in the electronic structure and charge density of GQD caused by the ionization of hydroxyl groups (Das G. S. et al., 2019). In the presence of salt (NaCl, up to 0.5M), IL-GQDs also exhibit high fluorescent stability (Supplementary Figure S3B in SI), indicating great potential in practical applications. Anti-photobleaching is crucial for fluorescent nanomaterials. The photostability of IL-GQDs in storing without protecting from room light for 30 days was studied. As shown in Supplementary Figure S3C (SI), the fluorescence intensity of IL-GQDs remains almost unchanged, indicating good stability in long-term storage. The ultraviolet lamp (365nm, 48W) was continuously irradiated to further study the anti-photobleaching properties of IL-GQDs. It is found that the fluorescence intensity remains 90% after continuous irradiation for 4h, suggesting that IL-GQDs have good anti-photobleaching characteristics (Supplementary Figure S3D in SI).

### Selective Fluorescence Quenching of IL-GQDs by S^2−^ Ion

**FIGURE 5:**
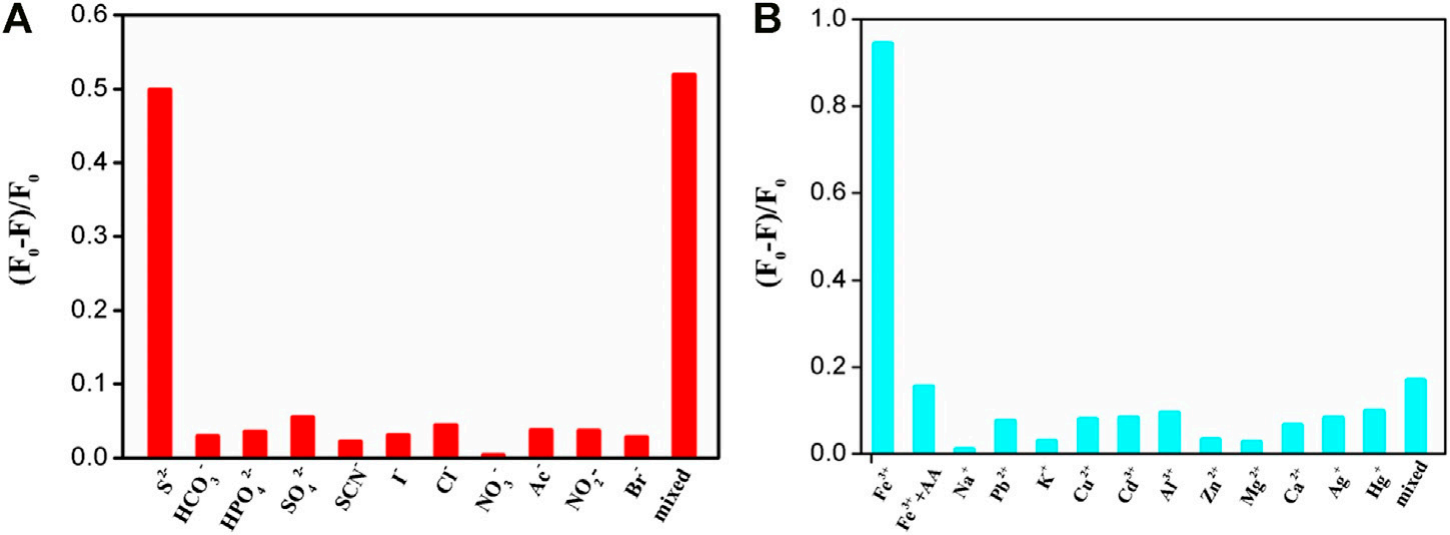
The relative fluorescent ratio of IL-GQDs in the presence of different single or mixed (the mixture of all ions on the *x* axis) anion **(A)** or cation. **(B)** AA (20mM) is added in case of mixed cations.

Due to the anion exchange ability of IL, the as-prepared IL-GQDs have great potential in the sensing of anion. The selectivity of IL-GQDs to different anions was investigated. As shown in Figure 5A, S^2−^ ion can strongly quench the fluorescence of IL-GQDs. In contrast, other physiologically or environmentally related anions have no significant effect on the fluorescence of IL-GQDs (Figure 5A). When multiple anions coexist, the fluorescence quenching rate is still close to that of single S^2−^ ion. As we all know, GQDs have been widely used as fluorescent probes in the sensing of metal cations. Therefore, the interaction between IL-GQDs and cautions was also investigated. As shown in Figure 5B, Fe^3+^ ions strongly quench the fluorescence of IL-GQDs, which may be attributed to the interaction between Fe^3+^ and -OH groups of GQDs (Shen et al., 2017). However, adding ascorbic acid (AA) can recover the Fe^3+^-quenched fluorescence of IL-GQDs(Luo et al., 2019). On the contrary, other single or mixed cations have no significant effects on the fluorescence intensity of the IL-GQDs (Figure 5B). In case of IL-free GQDs (Supplementary Figure S4 in SI), OH-GQDs have no selectivity toward S^2−^ ion. Thus, the anion selectivity of IL-GQDs results from the binding of IL. The fluorescence lifetime is applied to study the possible mechanism. Fluorescence lifetime of IL-GODs nanocomposite in the absence or presence of different concentrations of S^2−^ ion is investigated (Supplementary Figure S5 in SI). The fluorescence lifetime of IL-GODs slightly decreases with the addition of low concentration (1μM) of S^2−^ ion. With the increase of sulfur ion concentration (10μM or 50μM), the fluorescence lifetime of IL-GODs decreases significantly. The phenomena reveal the electron transfer between IL-GODs and S^2−^ ion, leading to fluorescence quenching.

### Detection of S^2−^ Ion Using IL-GQDs as Fluorescence Probe

**FIGURE 6:**
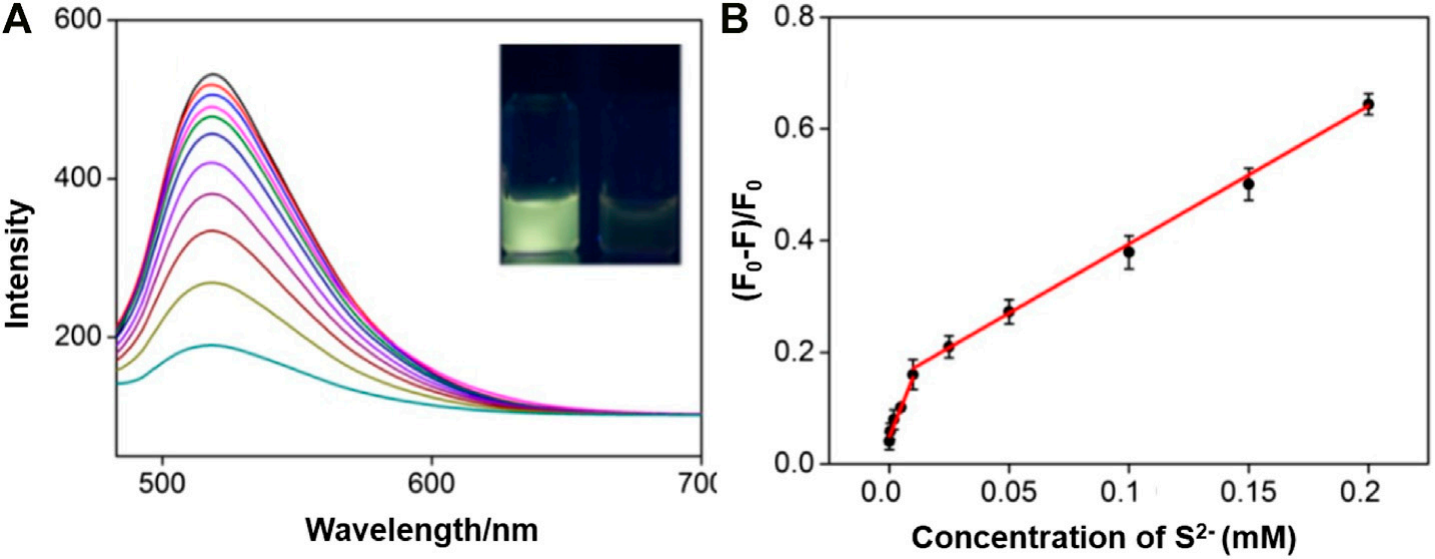
**(A)** FL emission spectra of IL-GQDs in the presence of different concentrations of S^2−^ ion. Inset images present the photographs of IL-GQDs solutions in the absence **(left)** or absence **(right)** of S^2−^ ion under 365nm UV light. **(B)** Linear relation curve between fluorescent quenching ratio and the concentration of sulfide ion.

Because of specific selectivity toward S^2−^ ions, IL-GQDs nanocomposite is applied as fluorescent probe to detect S^2−^ ion. In order to obtain the highest sensitivity, the detection conditions such as pH value and incubation time have been optimized. Depending on the pH of the solution, S^2−^ ion can exist in the form of dihydrogen sulfide (H_2_S), hydrogen sulfide anion (-SH), or as sulfide dianion. Thus, we only investigated the interaction between IL-GQDs and S^2−^ ion under near-neutral, neutral, or base conditions (pH 5–10) by calculating the relative fluorescence quenching ratio. As shown in Supplementary Figure S6A (SI), the highest fluorescence quenching is observed at pH 6. At the same time, fast kinetics is observed because the fluorescence quenching plateau appears, within 8min (Supplementary Figure S6B in SI). Under those optimal detection conditions, the fluorescent spectra of IL-GQDs for different concentration of S^2−^ ion are shown in Figure 6A. The linear detection range is from 100nM to 10μM (*R* ^2^ = 0.997) and 10μM to 0.2mM (*R* ^2^ = 0.991) (Figure 6B). When the signal-to-noise ratio is 3, the limit of detection is 23nM. Comparison between fluorescent detection of S^2−^ ion using different fluorescent probes is provided in Supplementary Table S1 (SI). In comparison with the fluorescent turn off–on mode that is based on the recovery of fluorescence mainly quenched by metal ions, our detection is simple and sensitive.

Finally, IL-GQDs nanocomposite is used as a fluorescence probe to detect S^2−^ ion in environmental samples (river water) (Table 1). A certain amount of S^2−^ ions is added to the samples, and the artificial ion concentration is determined by the standard addition method. The recovery rate is 96.9–102%, and the relative standard deviation (RSD) is no more than 2.0%. Compared with other methods for analyzing S^2−^ ion (e.g., ion chromatography), fluorescent sensor based on IL-GQDs has the advantages of simplicity, rapid detection, and low cost.

**TABLE 1:** Detection of S^2−^ in river water samples.

Sample	Concentration of S^2−^ (μΜ)		RSD (n = 3, %)	Recovery (%)
	Added	Found by IL-GQDs		
1	0.50	0.51	2.0	102.0
2	20.00	19.37	1.8	96.9
3	80.00	78.44	1.9	98.1

## Conclusion

In summary, we have proven that ionic liquid–modified graphene quantum dots (IL-GQDs) nanocomposite can directly and sensitively detect S^2−^ ion. The one-step bottom-up synthesis of IL-GQDs in aqueous solution is simple, green, mild, and of low cost. The hybridization of IL and GQDs makes GQDs have anion exchange capacity. The as-prepared IL-GQDs nanocomposite exhibits selectivity toward S^2−^ ion. Because GQDs have the characteristics of stable fluorescence, high selectivity, good biocompatibility, easy synthesis, and adjustable structure and properties, the possibility of direct detection of anion by IL-modified GQDs can be further expanded.

## Data Availability

The raw data supporting the conclusions of this article will be made available by the authors, without undue reservation.
