# Supplementary material for: Sensitive Detection of Sulfide Ion Based on Fluorescent Ionic Liquid–Graphene Quantum Dots Nanocomposite
Source: Front Chem. 2021 Apr 30;9:658045. doi: 10.3389/fchem.2021.658045 (PMC8120006; doi:10.3389/fchem.2021.658045)
Supplement: Supplementary file 1 [file DataSheet1.docx]

Supplementary Material

## Supplementary Scheme


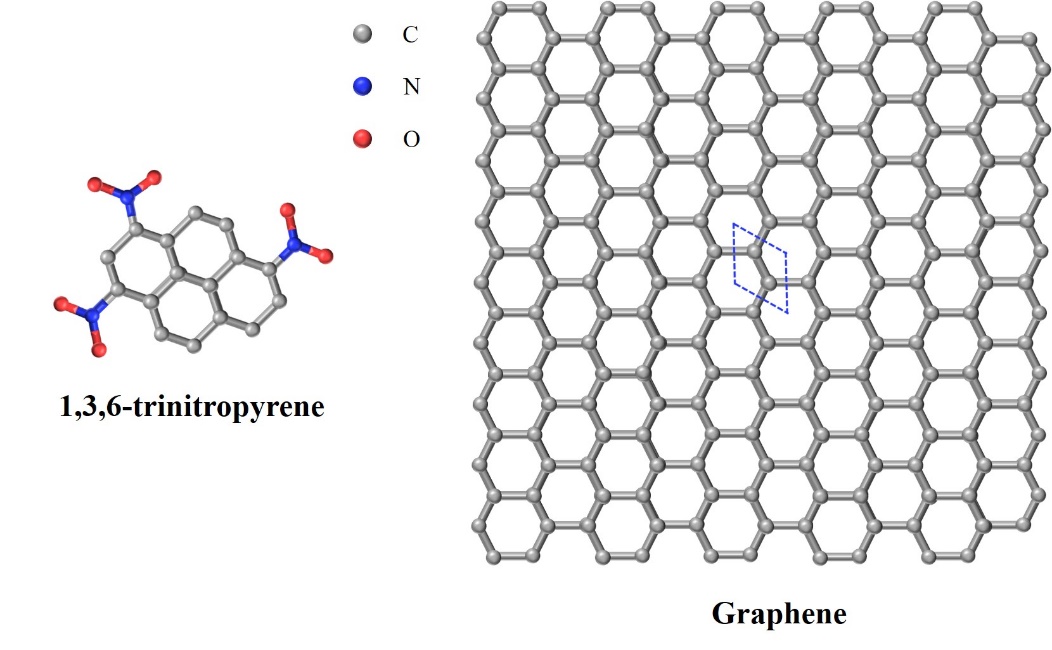


**Scheme 1** The structural demonstration of 1,3,6-trinitropyrene and graphene.

## Supplementary Figures


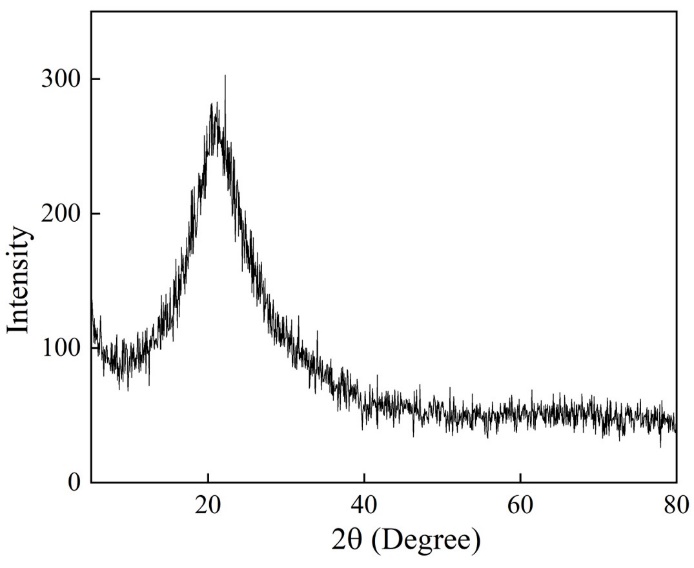


**Fig. S1** XRD patterns of IL-GQDs.


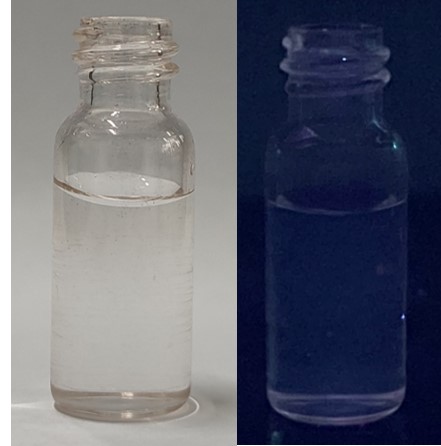


**Fig. S2** The photographs of control solution under visible (left) or 365 nm UV (right) light. The solution was obtained when only IL (1% v/v) was treated using the same preparation condition.


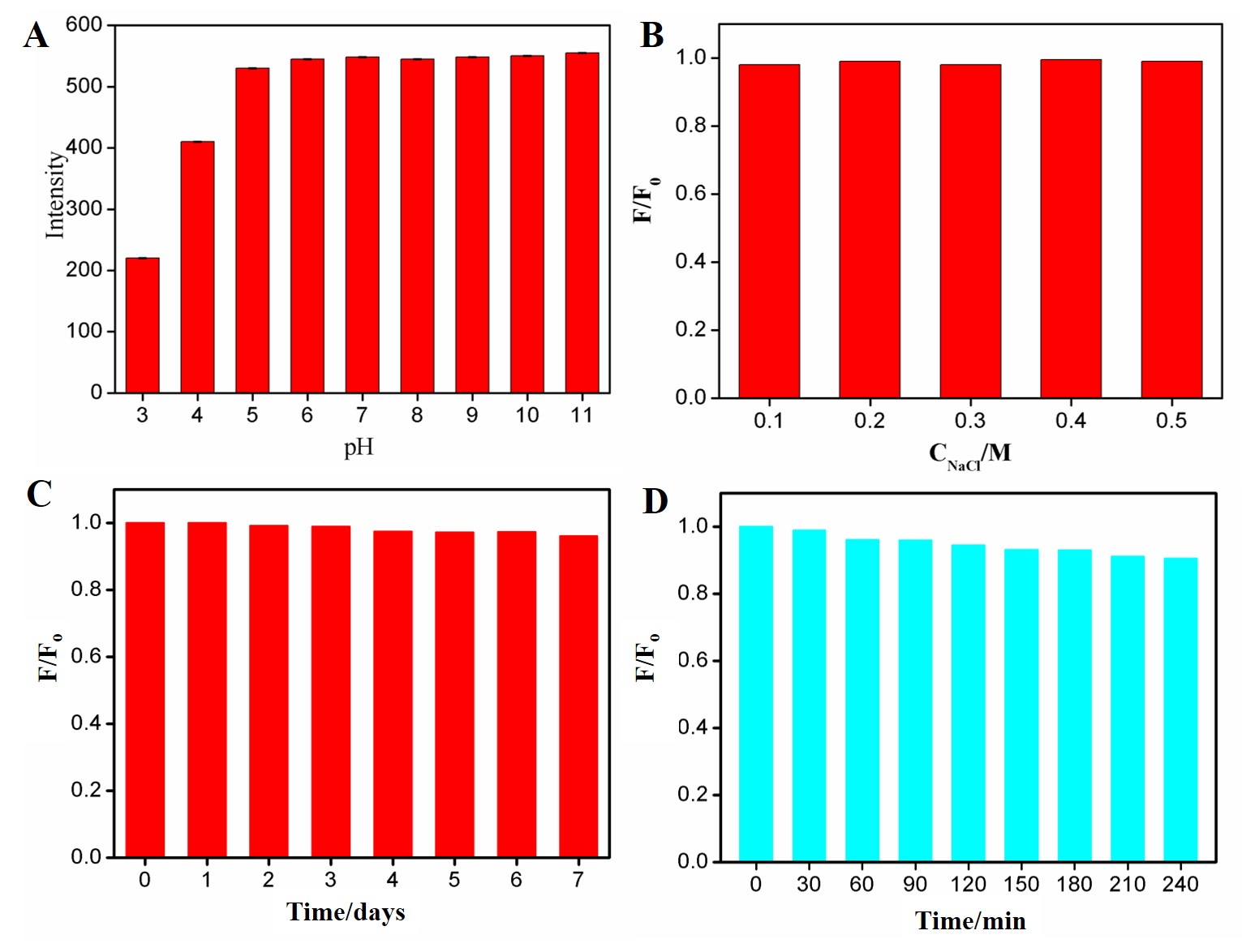


**Fig. S3** The FL intensity of IL-GQDs at different pH (A). The relative FL ratio of IL-GQDs under different concentration of NaCl (B), room temperature storage (C) and UV irradiation (365 nm, D).





**Fig. S4** The relative fluorescent ratio of the control OH-GQDs in presence of different anion.


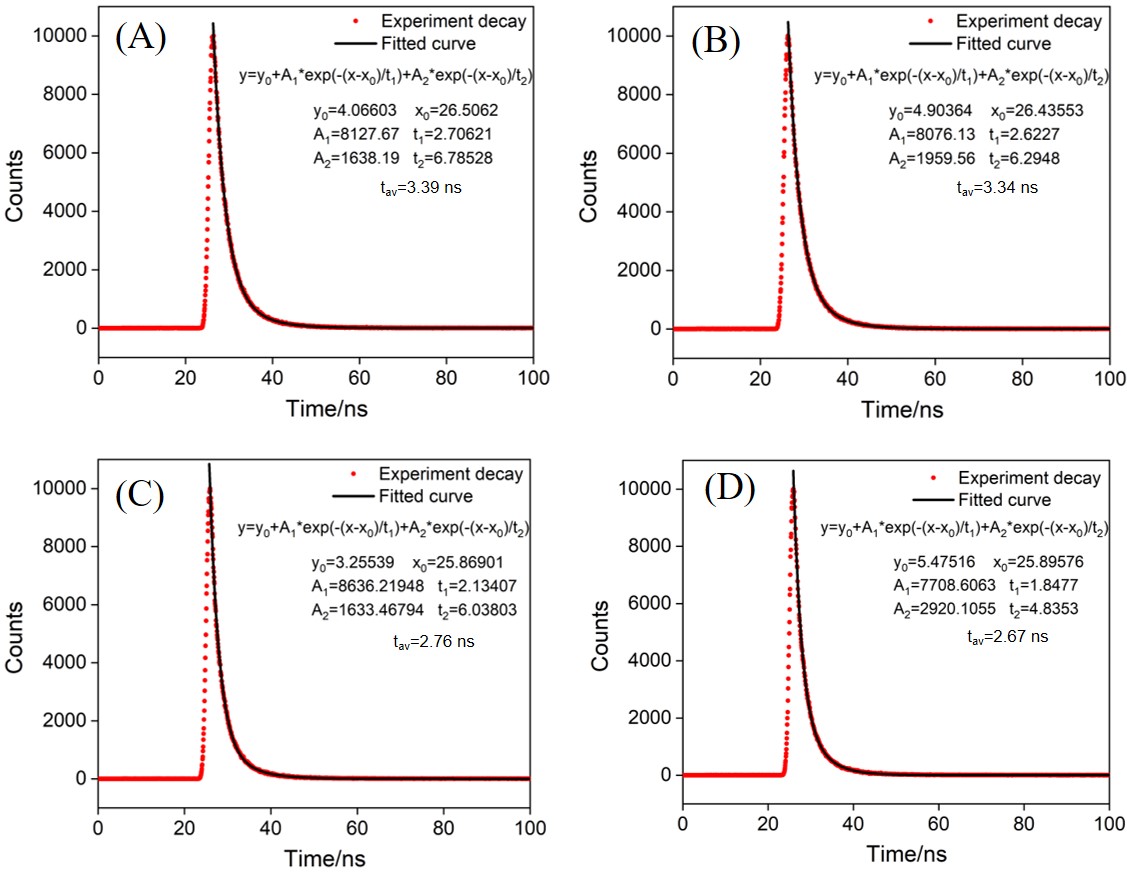


**Fig. S5** FL lifetime spectra of IL-GQDs in absence (A) or presence of 1 μM (B), 10 μM (C) or 50 μM (D) S^2-^ ion


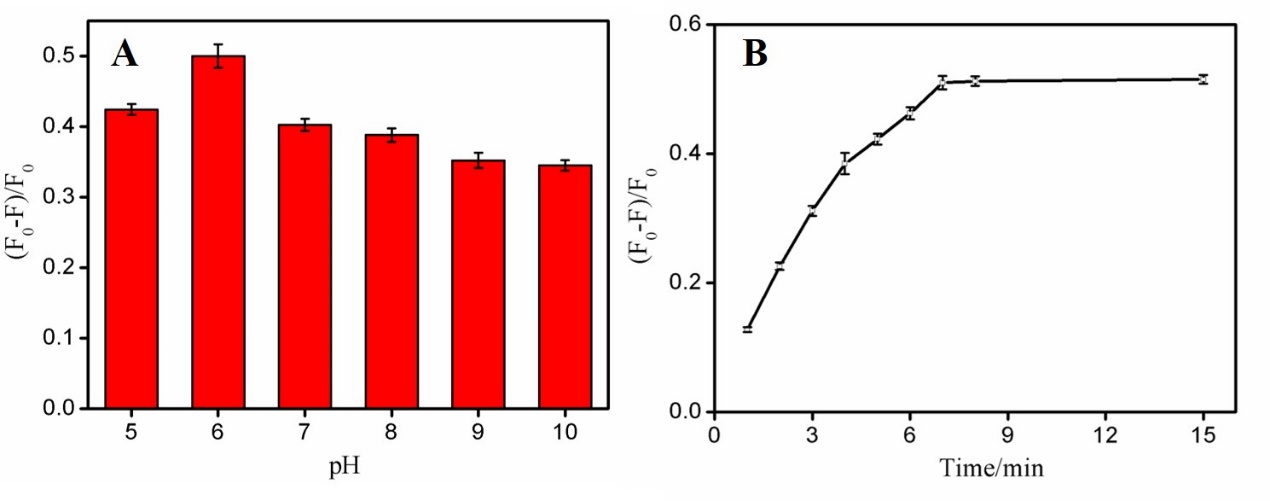


**Fig. S6** The effect of pH (A) and incubation time (B) on the fluorescent quenching ratio of IL-GQDs caused by S^2-^ ion.

## Supplementary Tables

Table S1 Comparison between fluorescent detection of S^2-^ ion using different fluorescent probe.

| ***fluorescent probe*** | ***mode*** | | ***Detection range*** | | ***LOD*** | | *Ref.* | | |  |  |
| --- | --- | --- | --- | --- | --- | --- | --- | --- | --- | --- | --- |
| *IL-GQD* | | FL turn off | | 100 nM - 10 μM  10 μM - 0.2 mM | | 23 nM | | | This work | | |
| *sulfanilic acid and glutathione functionalized GQDs* | | FL turn off-on | | 0-12 μM | | 20.3 nM | | 1 | | |  |
| *Graphitic carbon nitride QDs* | | FL turn off-on | | 0.2-21 μM | | 3.3 nM | | 2 | | |  |
| *N,S co-doped CDs* | | FL turn off-on | | 0.5-50 μM | | 83 nM | | 3 | | |  |
| *CDs and Au nanoparticles* | | FL turn off-on | | 0.03–0.6 μM | | 20 nM | | 4 | | |  |
| *quinoline-based organic ligand* | | FL turn off-on | | - | | 14.8 μM | | 5 | | |  |
| *Silver nanoparticles capped CDs* | | FL turn off-on | | 0.01–0.9 μM | | 0.01 μM | | 6 | | |  |
| *CDs and MnO_2_ nanoplate* | | FL turn off-on | | 2–25 μM | | 0.8 μM | | 7 | | |  |
| *Organic semiconductor polymer nanodots* | | FL turn off-on | | 1.25-75 μM | | 0.45 nM | | 8 | | |  |
| *fluorescent peptide* | | FL turn off-on | | 50 nM-1.0 mM | | 19 nM | | 9 | | |  |
| copper nanoclusters | | FL turn off-on | | 0-600 μM | | 26.3 nM | | 10 | | |  |

**References**

1. W.D. Na, Z.Y. Qu, X.Q. Chen, X.G. Su, A turn-on fluorescent probe for sensitive detection of sulfide anions and ascorbic acid by using sulfanilic acid and glutathione functionalized graphene quantum dots, *Sens. Actuators B Chem.*, 2018, **256**, 48-54.

2. X. Wang, X.F. Yang, N. Wang, J.J. Lv, H.J. Wang, M.M.F. Choi, W. Bian, Graphitic carbon nitride quantum dots as an "off-on" fluorescent switch for determination of mercury(II) and sulfide, *Microchim. Acta*, 2018, **185**, 471.

3. H. Wu, C. Tong, Nitrogen- and sulfur-codoped carbon dots for highly selective and sensitive fluorescent detection of Hg^2+^ ions and sulfide in environmental water samples, *J. Agric. Food Chem.* 2019, **67**, 2794-2800

4. N. Shahbazi, R. Zare-Dorabei, A novel “off-on” fluorescence nanosensor for sensitive determination of sulfide ions based on carbon quantum dots and gold nanoparticles: Central composite design optimization, *Microchem. J.* 2019, **145**, 996-1002.

5. J.M. Jung, J. H. Kang, J. Han, H. Lee, M.H. Lim, K. Kim,
C. Kim, A novel “off-on” type fluorescent chemosensor for detection of Zn2+
and its zinc complex for “on-off” fluorescent sensing of sulfide in
aqueous solution, in vitro and in vivo, *Sens. Actuators B Chem.*, 2018, **267**, 58-69.

6. B. Sinduja, S. A. John, Silver nanoparticles capped with carbon dots as a fluorescent probe for the highly sensitive Boff–on^ sensing of sulfide ions in water,
*Anal. Bioanal. Chem.* 2019, **411**, 2597-2605.

7. J. Liu, C. Liu, Z. Zhou, A turn-on fluorescent sulfide probe prepared from carbon
dots and MnO2 nanosheets, *Microchim. Acta*, 2019, **186**, 281.

8. C. Wang, J. Sun, H. Mei, F. Gao, Organic semiconductor polymer nanodots as a new kind
of off-on fluorescent probe for sulfide, *Microchim. Acta*, 2017, **184**, 445-451.

9. Y. Zhang,. Y. Cai, Y. He, Q. Lin, J. Ren, D. Cao, L. Zhang, A label-free fluorescent peptide probe for sensitive and selective determination of copper and sulfide
ions in aqueous systems, *RSC Adv.*, 2017, **11**, 7426-7435.

10. D. Wang, Z. Wang, X. Wang, X. Zhuang, C. Tian, F. Luan, X. Fu, Functionalized copper nanoclusters-based fluorescent probe with aggregation-induced emission property for selective detection of sulfide ions in food additives, *J. Agric. Food Chem.* 2020, **68**, 11301-11308.
